# Lack of replication of associations between multiple genetic polymorphisms and endurance athlete status in Japanese population

**DOI:** 10.14814/phy2.13003

**Published:** 2016-10-24

**Authors:** Thomas Yvert, Eri Miyamoto‐Mikami, Haruka Murakami, Motohiko Miyachi, Takashi Kawahara, Noriyuki Fuku

**Affiliations:** ^1^ Graduate School of Health and Sports ScienceJuntendo UniversityInzai‐cityChibaJapan; ^2^ Japan Society for the Promotion of ScienceChiyoda‐kuTokyoJapan; ^3^ Department of Sports and Life ScienceNational Institute of Fitness and Sports in KanoyaKanoya‐cityKagoshimaJapan; ^4^ Department of Health Promotion and ExerciseNational Institute of Health and NutritionNIBIOHNShinjuku‐kuTokyoJapan; ^5^ Medical CenterJapan Institute of Sports SciencesKita‐kuTokyoJapan

**Keywords:** Endurance runner, genotype score, physical performance, polymorphism

## Abstract

The aim of this study was to examine a polygenic profile related to endurance performance, based on current knowledge, in the Japanese population. We analyzed 21 genetic polymorphisms that have been reported to be associated with endurance performance and its related phenotypes in 175 endurance runners (60 international‐, 94 national‐, and 21 regional‐level) and 649 controls in the Japanese population. Then, we calculated the total genotype score (TGS) (maximum value of 100 for the theoretically optimum polygenic score) for endurance performance. There was no association between the TGS and endurance athlete status (Control: 49.0 ± 7.6, Regional: 47.3 ± 7.6, National: 49.1 ± 5.7, and International: 48.2 ± 7.0, *P *=* *0.626). These results suggested that TGSs based on the 21 previously published endurance performance‐associated polymorphisms do not influence endurance running performance in the Japanese population. Nevertheless, some marginal tendencies have to be noted: the frequencies of the *ACTN3* R577X rs1815739 RR+RX genotype and the *GNB3* rs5443 CC+CT genotype were higher in international athletes than in controls (85% vs. 73.6%, *P *=* *0.042 and 90% vs. 76%, *P *=* *0.007, respectively), but not significantly different after Bonferroni correction.

## Introduction

Elite athletic status is a complex trait resulting from the interaction of numerous factors including training methods, socio‐economic aspects, psychology, technology, injury history or diet, and genetic endowment is one of the many factors that affect athletic endurance performance. Many studies have attempted to identify genetic polymorphisms associated with elite endurance athlete status and physical performance traits such as maximum oxygen uptake, energetic metabolism, and muscle strength or mass (Ahmetov and Fedotovskaya 2015; Loos et al. 2015). The number of these potential polymorphisms is increasing each year, and it is now accepted that physical performance is highly polygenic (Miyamoto‐Mikami et al. 2016). Therefore, several studies have attempted to identify polygenic profiles that could affect the possibility to become an elite endurance athlete (Eynon et al. 2011; Ruiz et al. 2009; Williams et al. 2008). Nevertheless, most of these studies were conducted in Caucasian subjects. Since genetic background differs among different ethnicities, the effects of the previously studied polymorphisms on physical performance in Asian populations remain unclear. In particular, little information regarding polygenic profiles and endurance performance is available for Asian populations. Thus, the purpose of this study was to examine the association between a polygenic profile based on current knowledge and endurance athlete status in the Japanese population.

## Methods

### Subjects

This study included 175 Japanese endurance track‐and‐field athletes (65 women): 152 long‐distance runners (≥3000 m) and 23 middle‐distance runners (800–1500 m). The athletes were assigned to three groups according to their competitive achievement, as follows: (1) 60 international athletes, who participated at major international competitions, such as the Olympic Games or World and Asian Championships, and included several medalists at these international competitions; (2) 94 national athletes, who participated in Japanese national competitions; and (3) 21 regional athletes, with at least 3 years of competitive experience. The control group consisted of 649 nonathletic healthy Japanese (465 women) from the Tokyo area. Written informed consent was obtained from all subjects, and the study was approved by the ethics committees of the Juntendo University, the Japan Institute of Sports Sciences, and the National Institute of Health and Nutrition.

### Candidate gene polymorphisms

We searched for genetic polymorphisms that were associated with endurance performance‐related phenotypes (endurance performance, maximal oxygen consumption, lactate threshold, and trainability of these parameters) using PubMed.

Exclusion criteria were: (1) polymorphism presenting unknown or less than 5% minor‐allele frequency in Japanese populations; (2) presence of other polymorphism within two bases; (3) linkage disequilibrium with other target polymorphism; (4) unknown rs number; and (5) length polymorphisms. Finally, 22 polymorphisms were selected for the analysis (*PPARGCB* rs7732671 was excluded from analysis, not respecting Hardy–Weinberg equilibrium [HWE]); these are listed in Table 1.

**Table 1:** Studied polymorphisms for endurance performance

Gene symbol	Gene name	rs number	Polymorphism (Function)	Reference	Genotype score
Nuclear DNA					
*ACE*	angiotensin I converting enzyme	rs4340	I/D (Intron)	Myerson et al. (1999)	II = 2, ID = 1, DD = 0
*ACTN3*	actinin, alpha 3	rs1815739	C>T (Arg577Ter)	Yang et al. (2003)	TT = 2, CT = 1, CC = 0
*ADRA2A*	adrenoceptor alpha 2A	rs553668	T>C (3′‐UTR)	Wolfarth et al. (2000)	CC = 2, CT = 1, TT = 0
*ADRB2*	adrenoceptor beta 2	rs1042713	C>G (Gln27Glu)	Moore et al. (2001)	CC = 2, CG = 1, GG = 0
		rs1042714	A>G (Arg16Gly)	Wolfarth et al. (2007)	AA = 2, AG = 1, GG = 0
*ADRB3*	adrenoceptor beta 3	rs4994	T>C (Trp64Arg)	Santiago et al. (2011)	CC = 2, CT = 1, TT = 0
*APOE* 1	apolipoprotein E	rs429358	T>C (Cys112Arg)	Thompson et al. (2004)	E3E4 (E4E4,E4E2) = 2
		rs7412	C>T (Arg158Cys)		E3E2 (E2E2) = 1; E3E3 = 0
*CKM*	creatine kinase, muscle	rs8111989	A>G (3′‐near gene)	Rivera et al. (1997)	AA = 2, AG = 1, GG = 0
*COL5A1*	collagen, type V, alpha 1	rs12722	C>T (3′‐UTR)	Posthumus et al. (2011)	TT = 2, CT = 1, CC = 0
*GABPB1*	GA‐binding protein transcription factor, beta subunit 1	rs7181866	A>G (Intron)	Eynon et al. (2009)	GG = 2, AG = 1, AA = 0
*GNB3*	guanine nucleotide‐binding protein (G protein), beta polypeptide 3	rs5443	C>T (Synonymous)	Eynon et al. (2009)	TT = 2, CT = 1, CC = 0
*KDR*	kinase insert domain receptor	rs1870377	A>T (Gln472His)	Ahmetov et al. (2009a)	AA = 2, AT = 1, TT = 0
*NFATC4*	nuclear factor of activated T‐cells, cytoplasmic, calcineurin‐dependent 4	rs2229309	G>C (Gly160Ala)	Ahmetov et al. (2009b)	GG = 2, GC = 1, CC = 0
*PPARD*	peroxisome proliferator‐activated receptor delta	rs2016520	C>T (5′‐UTR)	Hautala et al. (2007)	TT = 2, CT = 1, CC = 0
*PPARGC1A*	peroxisome proliferator‐activated receptor gamma, coactivator 1 alpha	rs8192678	G>A (Gly482Ser)	Lucia et al. (2005)	GG = 2, AG = 1, AA = 0
*PPARGC1B*	peroxisome proliferator‐activated receptor gamma, coactivator 1 beta	rs7732671	G>C (Ala203Pro)	Ahmetov et al. (2009b)	not included
*SLC16A1*	solute carrier family 16 (monocarboxylate transporter), member 1	rs1049434	T>A (Asp490Glu)	Cupeiro et al. (2012)	AA = 2, AT = 1, TT = 0
*TFAM*	transcription factor A, mitochondrial	rs1937	G>C (Ser12Thr)	Ahmetov et al. (2009b)	CC = 2, GC = 1, GG = 0
*UCP2*	uncoupling protein 2	rs660339	C>T (Ala55Val)	Ahmetov et al. (2009b)	TT = 2, CT = 1, CC = 0
*UCP3*	uncoupling protein 3	rs1800849	C>T (5′‐UTR)	Ahmetov et al. (2009b)	TT = 2, CT = 1, CC = 0
Mitochondrial DNA					
*MT‐ND2*	mitochondrially encoded NADH dehydrogenase 2	m.4833 (Haplogroup G)	A>G (Thr122Ala)	Mikami et al. (2011)	G = 2, A = 0

Phenotype‐associated alleles in previous studies (optimal alleles) are underlined. Ala: alanine, Arg: arginine, Asp: aspartic acid, Cys: cysteine, Gln: glutamine, Glu: glutamic acid, Gly: glycine, His: histidine, Pro: proline, Ser: serine, Ter: termination codon, Thr: threonine, Trp: tryptophan, UTR: untranslated region, and Val: valine. ^1^GS of APOE was assigned by a combination of rs429358 and rs7412 genotypes based on Thompson et al. (2004) (PMID: 14767871): the E4 and E3 alleles, respectively, determined as “optimal” and “less optimal” for endurance phenotypes.

### Genotyping

Total DNA was isolated from venous blood or saliva, as previously described (Kikuchi et al. 2016). All polymorphisms were genotyped using TaqMan SNP Genotyping Assays and StepOnePlus^™^ Real‐Time PCR System (Applied Biosystems, Foster City, CA). Custom primers were used for the *MT‐ND2* polymorphism as follow: forward primer: 5‐GCCCCCTTTCACTTCTGAGT‐3; reverse primer: 5‐GGGCTAGTTTTTGTCATGTGAGAAG‐3; A‐allele probe, 5‐CAAGGCGCCCCTC‐3; G‐allele probe, 5‐CCAAGGCACCCCTC‐3. Genotype calling was conducted using StepOne^™^ Software v2.1 (Applied Biosystems, Foster City, CA). rs4341 being in complete linkage‐equilibrium with rs4340 in Asian populations (Tanaka et al. 2003), *ACE* I/D genotypes were calculated as follows: rs4341 G/G as D/D, C/G as I/D, and C/C as I/I.

### Total genotype score

Total genotype score (TGS) was calculated from the selected polymorphisms following the procedure previously described (Miyamoto‐Mikami et al. 2016; Williams et al. 2008). Each genotype was scored based on literature information (Table 1). We assigned a genotype score (GS) of 2, 1, and 0 to “optimal”, “intermediate”, and “less optimal” genotypes, respectively. Then, we summed the GSs and transformed the sum to a scale of 0–100 for easier interpretation. The TGS formula is as follows:

$$\begin{array}{cc} \text{TGS}= & ({\text{GS}}_{ACE}+{\text{GS}}_{ACTN3}+{\text{GS}}_{ADRA2A}+{\text{GS}}_{ADRB2}\\ & +{\text{GS}}_{ADRB3}+{\text{GS}}_{APOE}+{\text{GS}}_{CKM}+{\text{GS}}_{COL5A1}\\ & +{\text{GS}}_{GABPB1}+{\text{GS}}_{GNB3}+{\text{GS}}_{KDR}+{\text{GS}}_{NFATC4}\\ & +{\text{GS}}_{PPARD}+{\text{GS}}_{PPARGC1A}+{\text{GS}}_{SLC16A1}+{\text{GS}}_{TFAM}\\ & +{\text{GS}}_{UCP2}+{\text{GS}}_{UCP3}+{\text{GS}}_{TRHR}+{\text{GS}}_{VDR}) \end{array}$$

In the above formula, 40 is the result of multiplying 20 (number of analyzed polymorphisms) by 2, which is the score given to the optimal genotype.

### Statistical analysis

Hardy–Weinberg equilibrium was determined for each polymorphism by the *χ* ^2^ test. Genotypic association with elite athlete status was analyzed by logistic regression. Significance threshold was set after Bonferroni correction for multiple comparison at *P *<* *0.002 (=0.050/21). The group variances being unequal (Levene's test), Welch's one‐way ANOVA was used to compare means of TGSs among the four groups (control, regional, national, and international). All tests were performed using SNPstats software (http://bioinfo.iconcologia.net/SNPstats) (Solé et al. 2006) and the Statistical Package for Social Sciences (SPSS, v. 20. For Windows; SPSS Inc., Chicago, Illinois).

## Results

**Figure 1:**
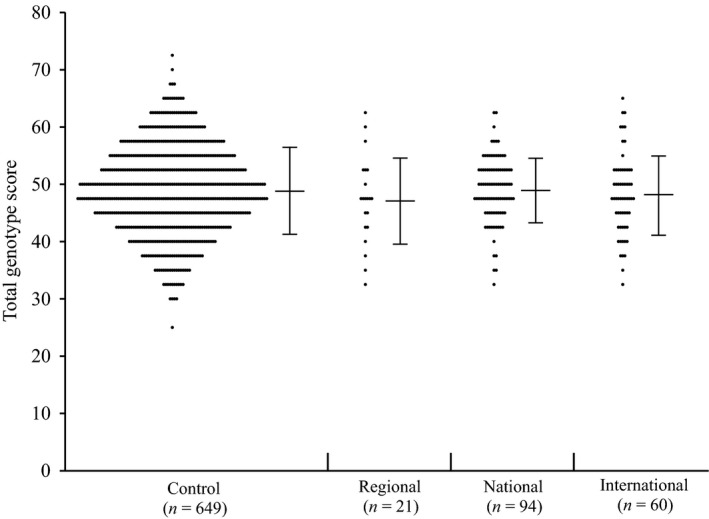
Total Genotype Score, based on 21 polymorphisms related with endurance performance, in the four studied groups (*P* = 0.626). The horizontal bars represent the mean values with standard deviations.

All the polymorphisms were in HWE, excepted *PPARGC1B* rs7732671 polymorphism, which was excluded from further analysis. No significant difference in TGSs was found among the four groups (Control: 49.0 ± 7.6, Regional: 47.3 ± 7.6, National: 49.1 ± 5.7, and International: 48.2 ± 7.0, *P *=* *0.626, Fig. 1). Even when the endurance athletes were divided into middle‐distance runners (Control: 49.0 ± 7.6, Regional: 45.8 ± 11.4, National: 48.4 ± 5.6, and International: 47.5 ± 8.1, *P *=* *0.871) and long‐distance runners (Control: 49.0 ± 7.6, Regional: 47.8 ± 6.0, National: 49.2 ± 5.7, and International: 48.3 ± 6.9, *P *=* *0.765), there were no significant differences in TGSs among the four groups. Furthermore, even when the endurance runners limited to the five outlier athletes who were world record holders and medalists at Olympics and/or World championships, their TGS were 47.1 ± 7.3 (Range: 35.0–57.5). Nevertheless, the polymorphisms *ACTN3* rs1815739 and *GNB3* rs5443 have been shown to be linked with international athlete status (Table 2). The frequencies of the *ACTN3* R577X rs1815739 CC+CT (i.e., RR+RX) genotypes and *GNB3* rs5443 CC+CT genotypes were higher in international athletes than in controls (85% vs. 73.6%, *P *=* *0.042 and 90% vs. 76%, *P *=* *0.007, respectively). However, after multiple testing corrections, the statistical significance of these polymorphisms was not retained (Adjusted *P* value: 0.882 for *ACTN3* rs1815739 and 0.147 for *GNB3* rs5443, respectively). All genotype frequencies data for 21 genetic polymorphism we analyzed were shown in Table 3.

**Table 2:** Allele and genotype frequencies of the polymorphisms presenting significant results between international athletes and controls

Gene symbol	Polymorphism rs number	Allele frequency					Genotype frequency, *n* (%)					International Athletes versus controls *P* value (OR [95% CI]) Genetic model^a^
		Allele	Control	Regional athlete	National athlete	International athlete	Genotype	Control	Regional athlete	National athlete	International athlete	
*ACTN3*	C/T rs1815739	C	0.47	0.43	0.48	0.56	CC	132 (20.3)	3 (13.4)	20 (21.3)	16 (26.7)	0.042 (2.03 [0.98–4.21]) C‐dominant
		T	0.53	0.57	0.52	0.44	CT	346 (53.3)	12 (57.1)	50 (53.2)	35 (58.3)	
							TT	171 (26.4)	6 (28.6)	24 (25.5)	9 (15.0)	
*GNB3*	C>T rs5443	C	0.51	0.38	0.56	0.59	CC	166 (25.6)	5 (23.8)	31 (33.3)	17 (28.3)	0.0072 (0.35 [0.15–0.83]) T‐recessive
		T	0.49	0.62	0.44	0.41	CT	327 (50.4)	6 (28.6)	42 (45.2)	37 (61.7)	
							TT	156 (24.0)	10 (47.6)	20 (21.5)	6 (10.0)	

a A most fitted genetic model based on Akaike information criterion is shown.

**Table 3:** Genotype frequencies of 20 polymorphisms in all groups

Gene symbol	Polymorphism (Function or location) rs number	Genotype	Genotype frequency, *n* (%)				
			Control	All athlete	Regional athlete	National athlete	International athlete
Nuclear DNA							
*ACE*	I/D (Intron) rs4340	II	269 (41.5)	72 (41.1)	7 (33.3)	39 (41.5)	26 (43.3)
		ID	301 (46.4)	78 (44.6)	11 (52.4)	40 (42.5)	27 (45.0)
		DD	79 (12.2)	25 (14.3)	3 (14.3)	15 (16.0)	7 (11.7)
*ACTN3*	C/T (Arg577Ter) rs1815739	CC	132 (20.3)	39 (22.3)	3 (14.3)	20 (21.3)	16 (26.7)
		CT	346 (53.3)	97 (55.4)	12 (57.1)	50 (53.2)	35 (58.3)
		TT	171 (26.4)	39 (22.3)	6 (28.6)	24 (25.5)	9 (15.0)
*ADRA2A*	T>C (3′‐UTR) rs553668	TT	112 (17.3)	34 (19.4)	5 (23.8)	16 (17.0)	13 (21.7)
		TC	326 (50.2)	80 (45.7)	10 (47.6)	41 (43.6)	29 (48.3)
		CC	211 (32.5)	61 (34.9)	6 (28.6)	37 (39.4)	18 (30.0)
*ADRB2*	A>G (Arg16Gly) rs1042713	AA	131 (20.2)	36 (20.6)	5 (23.8)	20 (21.3)	11 (18.3)
		AG	345 (53.2)	95 (54.3)	11 (52.4)	50 (53.2)	34 (56.7)
		GG	173 (26.7)	44 (25.1)	5 (23.8)	24 (25.5)	15 (25.0)
	C>G (Gln27Glu) rs1042714	CC	557 (85.8)	151 (86.3)	19 (90.5)	78 (83.0)	54 (90.0)
		CG	91 (14.0)	24 (13.7)	2 (9.5)	16 (17.0)	6 (10.0)
		GG	1 (0.2)	0 (0.0)	0 (0.0)	0 (0.0)	0 (0.0)
*ADRB3*	T>C (Trp64Arg) rs4994	TT	425 (65.5)	111 (63.4)	16 (76.2)	56 (59.6)	39 (65.0)
		TC	206 (31.7)	58 (33.1)	5 (23.8)	35 (37.2)	18 (30.0)
		CC	18 (2.8)	6 (3.4)	0 (0.0)	3 (3.2)	3 (5.0)
*APOE*	T>C (Cys112Arg) rs429358	TT	511 (78.7)	137 (78.3)	18 (85.7)	70 (74.5)	49 (81.7)
		TC	128 (19.7)	38 (21.7)	3 (14.3)	24 (25.5)	11 (18.3)
		CC	10 (1.5)	0 (0.0)	0 (0.0)	0 (0.0)	0 (0.0)
	C>T (Arg158Cys) rs7412	CC	597 (92.0)	161 (92.0)	17 (81.0)	89 (94.7)	55 (91.7)
		CT	51 (7.9)	14 (8.0)	4 (19.1)	5 (5.3)	5 (8.3)
		TT	1 (0.2)	0 (0.0)	0 (0.0)	0 (0.0)	0 (0.0)
*CKM*	A>G (3′‐near gene) rs8111989	AA	465 (71.7)	126 (72.0)	15 (71.4)	66 (70.2)	45 (75.0)
		AG	166 (25.6)	49 (28.0)	6 (28.6)	28 (29.8)	15 (25.0)
		GG	18 (2.8)	0 (0.0)	0 (0.0)	0 (0.0)	0 (0.0)
*COL5A1*	C>T (3′‐UTR) rs12722	CC	428 (66.0)	132 (75.4)	18 (85.7)	73 (77.7)	41 (68.3)
		CT	204 (31.4)	39 (22.3)	3 (14.3)	20 (21.3)	16 (26.7)
		TT	17 (2.6)	4 (2.3)	0 (0.0)	1 (1.1)	3 (5.0)
*GABPB1*	A>G (Intron) rs7181866	AA	391 (60.2)	102 (58.3)	13 (61.9)	54 (57.5)	35 (58.3)
		AG	226 (34.8)	60 (34.3)	7 (33.3)	33 (35.1)	20 (33.3)
		GG	32 (4.9)	13 (7.4)	1 (4.8)	7 (7.5)	5 (8.3)
*GNB3*	C>T (Synonymous) rs5443	CC	166 (25.6)	53 (30.5)	5 (23.8)	31 (33.3)	17 (28.3)
		CT	327 (50.4)	85 (48.9)	6 (28.6)	42 (45.2)	37 (61.7)
		TT	156 (24.0)	36 (20.7)	10 (47.6)	20 (21.5)	6 (10.0)
*KDR*	A>T (Gln472His) rs1870377	AA	229 (35.3)	60 (34.3)	7 (33.3)	36 (38.3)	17 (28.3)
		AT	303 (46.7)	83 (47.4)	12 (57.1)	35 (37.2)	36 (60.0)
		TT	117 (18.0)	32 (18.3)	2 (9.5)	23 (24.5)	7 (11.7)
*NFATC4*	G>C (Gly160Ala) rs2229309	GG	447 (68.9)	120 (68.6)	10 (47.6)	65 (69.2)	45 (75.0)
		GC	181 (27.9)	48 (27.4)	11 (52.4)	25 (26.6)	12 (20.0)
		CC	21 (3.2)	7 (4.0)	0 (0.0)	4 (4.3)	3 (5.0)
*PPARD*	C>T (5′‐UTR) rs2016520	CC	29 (4.5)	5 (2.9)	0 (0.0)	3 (3.2)	2 (3.3)
		CT	203 (31.3)	58 (33.1)	7 (33.3)	33 (35.1)	18 (30.0)
		TT	417 (64.2)	112 (64.0)	14 (66.7)	58 (61.7)	40 (66.7)
*PPARGC1A*	G>A (Gly482Ser) rs8192678	GG	191 (29.4)	45 (25.7)	6 (28.6)	27 (28.7)	12 (20.0)
		GA	324 (49.9)	87 (49.7)	10 (47.6)	46 (48.9)	31 (51.7)
		AA	134 (20.6)	43 (24.6)	5 (23.8)	21 (22.3)	17 (28.3)
*SLC16A1*	T>A (Asp490Glu) rs1049434	TT	61 (9.4)	24 (13.7)	2 (9.5)	15 (16.0)	7 (11.7)
		TA	300 (46.2)	75 (42.9)	10 (47.6)	41 (43.6)	24 (40)
		AA	288 (44.4)	76 (43.4)	9 (42.9)	38 (40.4)	29 (48.3)
*TFAM*	G>C (Ser12Thr) rs1937	GG	420 (64.7)	115 (65.7)	15 (71.4)	56 (59.6)	44 (73.3)
		GC	207 (31.9)	53 (30.3)	6 (28.6)	32 (34.0)	15 (25.0)
		CC	22 (3.4)	7 (4.0)	0 (0.0)	6 (6.4)	1 (1.7)
*UCP2*	C>T (Ala55Val) rs660339	CC	165 (25.4)	43 (24.6)	9 (42.9)	21 (22.3)	13 (21.7)
		CT	346 (53.3)	82 (46.9)	7 (33.3)	46 (48.9)	29 (48.3)
		TT	138 (21.3)	50 (28.6)	5 (23.8)	27 (28.7)	18 (30.0)
*UCP3*	C>T (5′‐UTR) rs1800849	CC	334 (51.5)	81 (46.3)	13 (61.9)	42 (44.7)	26 (43.3)
		CT	257 (39.6)	82 (46.9)	7 (33.3)	45 (47.9)	30 (50.0)
		TT	58 (8.9)	12 (6.9)	1 (4.8)	7 (7.5)	4 (6.7)
Mitochondrial DNA							
*MT‐ND2*	A>G (Thr122Ala) m.4833	A	594 (91.5)	163 (93.1)	20 (95.2)	86 (91.5)	57 (95.0)
		G	55 (8.5)	12 (6.9)	1 (4.8)	8 (8.5)	3 (5.0)

Gene names of the gene symbols are show in Table 1. All athletes comprise regional‐, national‐, and international‐level athletes.

## Discussion

In this study, we observed that mean values of endurance TGS, based on 21 candidates polymorphisms, did not differ between elite Japanese endurance runners and controls (Fig. 1). A possible explanation of our lack of significance could be that most of the polymorphisms included in our TGS were reported to be associated with endurance performance in Caucasian populations, and it is acknowledged that differences exist in genotype frequencies and haplotype networks between ethnic groups. For example, it has recently been found in East‐Asian athletes that the *ACE* I/D alleles were associated with elite athlete status, in opposition with the results generally obtained in Caucasian athletes (Wang et al. 2013). Therefore, it is conceivable that the present TGS included, besides *ACE* I/D, polymorphisms that could also present associations of opposite direction in Asian populations. Furthermore, based on the present findings in controls, the chances of finding a Japanese individual with a “theoretically” perfect TGS was 9.0 × 10^−13^. Of course, our lack of significant results could also be explained by statistical errors. Furthermore, functional significance of most of the polymorphisms analyzed remains unclear; therefore, we cannot exclude the possibility that our TGS included polymorphisms that do not influence endurance performance. In addition, it is possible that the studied polymorphisms affect the relevant physiology differently in Caucasian and Japanese populations owing to differences in environmental factors, such as training methods. Furthermore, our genotype score gave all genotypes the same weight; this may not be a true effect of the physiologic/biologic basis of athlete status. We also did not examine interactions among genes and/or between genes and environment that might affect elite athlete status, because sample size is not enough in this study. Thus, in future, extensive studies are required to consider environmental factors and gene–environment interactions as well as gene–gene interactions.

Two of the studied polymorphisms, namely *ACTN3* rs1815739 and *GNB3* rs5443, were individually linked with elite endurance athlete status (Table 2), although the statistical significances were not confirmed after multiple‐testing corrections. The frequency of *ACTN3* 577XX genotype was under‐represented in international athletes, compared with controls. *α*‐actinin‐3 is almost exclusively expressed in fast‐twitch muscle fibers, where it acts as a lattice structure that anchors actin‐containing thin filaments; this stabilizes the muscle contractile apparatus, thereby conferring a higher capacity for force absorption/transmission compared with slow fibers. Originally, it was thought that the XX genotype presented an advantage for endurance performance. However, considering the loss of functionality due to the XX genotype (Lee et al. 2016), it is presently thought that the R allele and the presence of *α*‐actinin‐3 in fast‐twitch muscle fibers may be beneficial also to endurance performance (Lee et al. 2016; Kikuchi et al. 2016); this is in accordance with our results.

We also found a possible relation between the *GNB3* rs5443 polymorphism and international endurance athlete status. The *GNB3* gene encodes the beta subunit of heterotrimeric guanine nucleotide‐binding proteins (G protein), which integrate signals between receptors and effector proteins. It is thought to confer an advantage on endurance performance, enhancing glycogen and fatty acid metabolism through the cAMP‐insulin receptor pathway (Eynon et al. 2009). Eynon et al. (Eynon et al. 2009) found that the TT genotype frequency was significantly higher in elite Israeli endurance athletes than in controls or sprinters. Our results showed a tendency in the opposite direction: the C allele frequency was higher in international endurance athletes than in controls. However, when Ruiz et al. (2011) conducted a replication of the Eynon et al. study in larger cohorts and other ethnicities (Israeli and Spanish), they could not find significant associations. As we mentioned above, there are several possible explanations justifying these results inconsistency (e.g., ethnicity differences, statistical errors, and/or environmental interactions).

## Practical Applications

Understanding the genetic of athletic performance is an important point in the development of future methods for talent identification in sport. Obtained data here suggest that the selected multiple genetic effect is not related to endurance performance in Japanese runners, so this fact should be taken into account in the future, especially for Asian athletes. Our nonsignificant results for being an elite runner based on the studied polymorphisms confirm that the possibility of becoming an elite athlete depends on numerous influential factors.

## Conclusions

In conclusion, our TGS based on 20 polymorphisms related with endurance performance (and related phenotypes), mostly in Caucasian populations, has not been found to be associated with elite endurance athlete status in the Japanese population. These results suggest that most of the polymorphisms analyzed in this study may not influence endurance athlete status in Japanese runners, with the exception of the *ACTN3* rs1815739 and *GNB3* rs5443 polymorphisms. In order to identify polygenic profiles that allow us to distinguish the potential of someone in the Japanese population to become an international athlete, it seems that future studies should further focus on polymorphisms for which associations have been observed with elite athlete status in Asian populations, and with robust replications.

## Conflict of Interest

None declared.
